# An ethics curriculum for short-term global health trainees

**DOI:** 10.1186/1744-8603-9-5

**Published:** 2013-02-14

**Authors:** Matthew DeCamp, Joce Rodriguez, Shelby Hecht, Michele Barry, Jeremy Sugarman

**Affiliations:** 1 Johns Hopkins Berman Institute of Bioethics and Division of General Internal Medicine, 1809 Ashland Avenue, Baltimore, MD, 21205, USA; 2 Center for Innovation in Global Health, Stanford University and School of Medicine, 291 Campus Drive, Room LK3CO2, MC: 5216, Stanford, CA, 94305-5119, USA; 3 Johns Hopkins University, Mason Hall, Baltimore, USA; 4 Harvey M. Meyerhoff Professor of Bioethics and Medicine, Johns Hopkins Berman Institute of Bioethics, and Division of General Internal Medicine, 1809 Ashland Ave, Baltimore, MD, 21205, USA

**Keywords:** Curriculum development, Ethics, Global health education, Global health electives, Global health training, Online education, Short-term medical outreach, Evaluation

## Abstract

**Background:**

Interest in short-term global health training and service programs continues to grow, yet they can be associated with a variety of ethical issues for which trainees or others with limited global health experience may not be prepared to address. Therefore, there is a clear need for educational interventions concerning these ethical issues.

**Methods:**

We developed and evaluated an introductory curriculum, “Ethical Challenges in Short-term Global Health Training.” The curriculum was developed through solicitation of actual ethical issues experienced by trainees and program leaders; content drafting; and external content review. It was then evaluated from November 1, 2011, through July 1, 2012, by analyzing web usage data and by conducting user surveys. The survey included basic demographic data; prior experience in global health and global health ethics; and assessment of cases within the curriculum.

**Results:**

The ten case curriculum is freely available at http://ethicsandglobalhealth.org. An average of 238 unique visitors accessed the site each month (standard deviation, 19). Of users who had been abroad before for global health training or service, only 31% reported prior ethics training related to short-term work. Most users (62%) reported accessing the site via personal referral or their training program; however, a significant number (28%) reported finding the site via web search, and 8% discovered it via web links. Users represented different fields: medicine (46%), public health (15%), and nursing (11%) were most common. All cases in the curriculum were evaluated favorably.

**Conclusions:**

The curriculum is meeting a critical need for an introduction to the ethical issues in short-term global health training. Future work will integrate this curriculum within more comprehensive curricula for global health and evaluate specific knowledge and behavioral effects, including at training sites abroad.

## Background

Surveys of medical students [1,2] and residents in varying specialties [3-6] demonstrate widespread and increasing interest in global health training electives abroad. These electives promote a number of goals for trainees including the acquisition of global health knowledge, refinement of clinical skills, development of cultural sensitivity, and cultivation of social justice [7-10]. Evidence suggests that global health electives might support residents’ fulfillment of certain education requirements (e.g., the U.S. Accreditation Council for Graduate Medical Education [11]) and lead trainees to pursue careers in underserved areas [12,13]. When done well, short-term electives can also contribute to greater global health equity by supporting long-term collaborative efforts [14].

Training electives across international borders can raise a number of ethical issues, including lack of adequate supervision, exceeding trainees’ level of training, sustainability of benefits, and reducing the risk of harm, among others [9,15-19]. Ethical issues can also arise when trainees engage in research, for example, when dealing with the challenge of obtaining informed consent [20]. Broad awareness of these ethical issues began with anecdotes, personal narratives, and case studies of trainees struggling with ethical issues abroad [21-25]. These issues are now being examined with systematic qualitative methods involving trainees [26-28] and faculty [29].

The need to address ethical issues in global health training has also been recognized in proposed ethics guidelines [30-33]. Some program planners have proposed improving current short-term global health training programs by acknowledging and managing ethical issues, either explicitly [34-36] or implicitly [37,38]. A recent international collaborative effort, the Working Group on Ethics Guidelines for Global Health Training (WEIGHT), developed best practice guidelines for training experiences in global health [39]. The WEIGHT guidelines address a wide range of ethical issues faced by trainees, host institutions, and sending institutions to ensure mutual and reciprocal benefits for all stakeholders. Among a number of key issues, the guidelines emphasize the need for full accounting of costs associated with short-term training, the importance of long-term partnerships, and the need for adequate supervision and preparation of trainees.

However, a recent literature review found infrequent inclusion of ethics or social responsibility among key competencies for undergraduate or graduate global health education [40], and a clear need exists for an accessible, introductory ethics curriculum geared toward trainees. In this article, we describe the development of a freely available, introductory online ethics curriculum, “Ethical Challenges in Short-term Global Health Training” (http://ethicsandglobalhealth.org). We then report usage statistics and demographic data of curriculum users, including information about prior global health and global health ethics experiences, to assess the curriculum’s ability to reach its target audience. In closing we discuss the implications of these findings for future ethics curriculum development for short-term global health training. We focus on trainees in the traditional sense (i.e., medical professionals still in training), where a unique opportunity exists to introduce these issues. More broadly, however, our use of “trainees” could include individuals, including faculty and independent practitioners, with limited or no prior global health experience. We have a similarly broad definition of “short-term training and service programs,” recognizing that no universal definition of key terms, such as “short-term” or “training,” may exist and that diverse programs might benefit from an introductory ethics curriculum.

## Methods

We developed the curriculum in four stages: (1) case solicitation; (2) content drafting; (3) content review; and (4) curriculum launch. This was followed by an open user evaluation to investigate the curriculum’s ability to reach target users and inform future curriculum development. The primary objective of the curriculum was to increase awareness of common ethical issues trainees might face in short-term global health training and service programs. Secondary objectives included trainee acquisition of strategies for dealing with these issues and increased trainee confidence in navigating them.

### Case solicitation

The authors solicited actual ethical issues experienced by trainees and program leaders within short-term programs from members of WEIGHT; program leaders from universities in the Consortium of Universities for Global Health (CUGH); colleagues who administer training programs; cases cited in the academic literature; and personal experiences. WEIGHT included a number of members from low- or middle-income countries (LMIC) worldwide, and CUGH membership similarly includes universities located in LMICs. After collecting a number of ethical issues and scenarios with varying ethics themes, we employed a purposive strategy to develop the received issues and scenarios into ten cases meant to address a range of important and commonly encountered ethical issues. Disagreements were resolved by consensus.

### Content drafting

For each case, we chose three major ethical themes or issues to highlight. To illuminate each theme, a primary author scripted a short video vignette and a thought provoking multiple-choice question with corrective feedback for both correct and incorrect answers. Each case included trainees at various stages of professional development to engage the curriculum’s target audience. The primary author additionally drafted a conclusion page to summarize the themes and provided additional relevant references and resources. For all cases, identifying locales, persons, and institutions were removed. Because real-life cases are rich in detail but might not reveal all three important themes, some elements of different cases were combined or fictionalized to better meet the educational objectives.

### Content review

**Figure 1:**
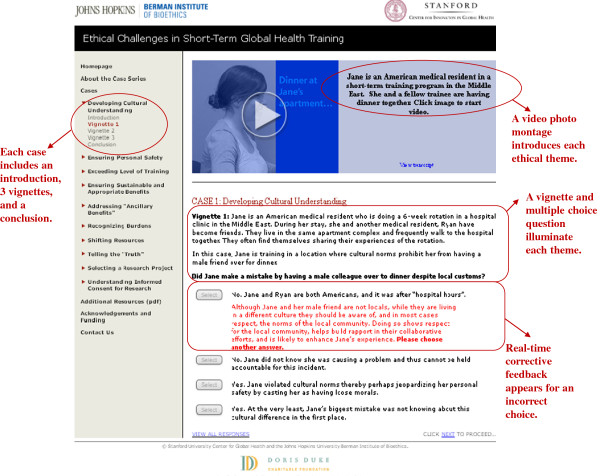
Representative screenshot of a case, demonstrating key features of the online curriculum.

After drafting each case, we reviewed and edited the content internally. Following internal review, video vignettes were filmed with volunteers, many of whom were from the countries or regions depicted. Content was translated into web format with technical assistance from Twisted Ladder Media™ and evaluated for accuracy by the authors. The anatomy of the cases, using a case screen shot as an example, is depicted in Figure 1. External content review, including review of questions and correct answers, was then solicited from members of WEIGHT and colleagues in bioethics. This helped ensure the curriculum avoided unjustified strong viewpoints on overly complex or controversial issues, even while it intends to raise awareness of them. In summer 2011, global health fellows through Stanford University’s Center for Innovation in Global Health pilot tested the curriculum. These fellows, most of whom were medical residents preparing for a short-term experience abroad, represented one target audience for the curriculum. At each stage suggestions for clarification and improvement were incorporated into the curriculum.

### Curriculum launch

The curriculum launched November 1, 2011. Since the target audience included trainees from diverse disciplines with limited or no experience in global health planning to travel abroad for short-term training, the curriculum was publicized in a number of ways, including: posting on the Johns Hopkins Berman Institute of Bioethics and Stanford University Center for Innovation in Global Health web pages (including Facebook and Twitter); communication via email lists, such as through the Consortium of Universities for Global Health, Global Health Education Consortium, and American Medical Student Association; and via direct personal communication to colleagues in global health.

### Open user evaluation

To assess the curriculum’s ability to reach its target users and obtain feedback on the curriculum content, we monitored web use statistics and requested users to complete anonymous surveys, which we had developed (described in more detail below).

Web statistics were provided by the web host and allowed tracking of the use of the curriculum and referral patterns important for understanding curriculum dissemination. First, to assess overall traffic, we collected hits, visits, and unique visits to the site. When a user accesses any site content (e.g., a video montage), a “hit” is recorded. If the user navigates several pages within a specified time (i.e., thirty minutes), a “visit” is recorded. If that user’s IP address has not been recorded previously within a specified time (i.e., thirty minutes), it counts as a “unique visit.” To approximate unique users, we were most interested in unique visits. This measure is important because web crawlers and other automated programs randomly access online content and may inflate the number of hits. Second, to assess how users access the site, the web host tracked the website from which a user came to visit our site (i.e., the referrer). This includes “direct referrals” that occur when a user directly types in the web link, accesses it from an email or other document, or uses bookmarks within their browser. Some web crawlers are also recorded as direct referrals.

The anonymous survey – conducted using Survey Monkey™ – was accessible from various points within the curriculum. The basic user survey included demographic data (e.g., age, sex, race, ethnicity, citizenship, and occupation) and prior experience in global health and global health ethics. In addition, users were separately asked to complete a brief survey following each case using 5-point Likert scales (from “strongly disagree” to “strongly agree”), yes/no questions, and open ended feedback. Data were downloaded in Excel and descriptively analyzed. This portion of the research was declared exempt from further review by the Johns Hopkins Medicine Institutional Review Board. The surveys are provided in Additional files 1 and 2.

## Results

### The curriculum

The ten cases remain available at http://ethicsandglobalhealth.org and address ethical issues in three different domains:

Trainee Behavior

1. Developing Cultural Understanding

2. Ensuring Personal Safety

3. Exceeding Level of Training

4. Telling the “Truth”

Broader Context of Short-term Programs

5 Ensuring Sustainable and Appropriate Benefits

6. Addressing “Ancillary Benefits”

7. Recognizing Burdens

8. Shifting Resources

Research

9. Selecting a Research Project

10. Understanding Informed Consent

While the responsibilities and decisions of trainees, host sites, sending institutions, and sponsors in short-term global health training overlap and intersect, the curriculum’s focus is on trainees. As such, some cases focus directly on trainee behavior, such as “Exceeding Level of Training” or “Telling the Truth.” Others place the trainee in complex situations where host site and sending institution responsibilities affect trainees’ experiences, such as in “Ensuring Sustainable and Appropriate Benefits” or “Recognizing Burdens.” The final two cases address difficult research questions trainees might face. Each case requires ten minutes or less to complete (as determined by pilot users), and all ten cases follow a similar structure, can be completed in any order, and do not require a login or email account for access. The certificate of completion for each case allows instructors to use any or all of the cases for particular needs and verify that learners have completed the case (e.g., prior to a class discussion).

For example, describing case four, “Ensuring Sustainable and Appropriate Benefits,” illustrates how the curriculum uses video montages, multiple choice questions, and real-time corrective feedback to meet curriculum objectives. In this case, a fifteen second video clip (with transcript available for slow Internet speeds) depicts a medical trainee with the mother of a sick child. The trainee is struggling with whether to give the sick child the only antibiotic on hand, even though it represents substandard treatment. In the first multiple-choice question, the user must decide whether to give the antibiotic. If the user incorrectly chooses, “Yes. Anything might help,” red text corrects the user and asks him or her to choose again. The correct answer asks the trainee to consult with his or her supervisor first and acknowledges the complexity of a single “correct” answer in this case. Thus, vignette one introduces trainees to an important ethical theme in short-term training, the potential for limited resources.

The second vignette builds upon this. In this video clip, the trainee is taking the right approach and discussing the matter with his supervisor. The trainee asks, “Why do we only have this particular antibiotic?” After his supervisor tells him it was the only one donated, the multiple-choice question forces the user to critically examine ethical issues arising with donated items. Concepts include the need for community involvement in decision-making and assurance that the items truly respond to local community needs. Thus, vignette two introduces trainees to another important theme in short-term programs, ethical issues with donated medical supplies.

Following a third vignette, the Conclusion provides a short framework for trainees might use to explore the proposed benefits of short-term programs:

*Who* decided the benefits were needed?

*What* counts as a benefit of a short-term program in the first place?

*Where* is the benefit distributed (i.e., how was this site chosen)?

*How* are benefits distributed?

And, are the benefits *sustainable*?

The conclusion also provides a reference and link to the World Health Organization’s *Guidelines for Drug Donations* for further reading.

### Web usage data

Web data of usage were collected for eight months (November 1, 2011, through July 1, 2012). Since launching the curriculum, the number of unique visits per month has been nearly stable, with a mean of 238 per month and a standard deviation of 19. No month had more than 300 unique visitors, and no month had less than 200.

During this eight month period, the top referrer to our site was a “direct referrer,” which as stated previously, represents when a user directly types in the web link, accesses it from an email or other document, or uses bookmarks within a web browser (as well as activity from some web crawlers). Direct referrers represented more than 30% of activity (38,097 referrals). No other referrer represented more than 1% of total hits as a result of web crawler activity, but the results were nonetheless revealing: After direct referrals, the next three top referrers were Google (273 referrals, where users presumably access the site from a Google search page), the Stanford Center for Innovation in Global Health (126 referrals); and the Johns Hopkins Berman Institute of Bioethics (66 referrals). The latter two sites contain links to the curriculum. While not statistically significant, the number of Google referrals appeared to increase over time, from 10 in November 2011 to 82 in June 2012.

### Open user data

The Open Evaluation data results included demographic data of course users; prior global health and global health ethics experience; and assessment of individual cases.

Table 1 presents users’ demographic data. The nature of open recruitment prevents calculation of a completion rate; however, the 158 respondents represented 8% of total unique visits. Most open users were female (69%), with a mean age of approximately 37 years. More than one-quarter of users reported being non-U.S. citizens. The most frequently reported non-U.S. citizenships were Canadian (9), Nigerian (5), and Indian (5). In total, 22 different non-U.S. citizenships were reported. About half of the users reported that they were “already practicing” in their field (i.e., not currently pursuing a degree). Data regarding reported fields of practice are also presented in Table 1, with medicine the most frequent (46%), followed by public health (15%) and nursing (11%).

**Table 1:** Demographic data for users from November 1, 2011, through July 1, 2012 (total N = 158, users do not have to answer all questions)

	**Users (%)**
**Female**	109/158 (69%)
**Mean age (SD), years**	37 (14)
**Race/Ethnicity**	
American Indian/Alaska Native	1/158 (1%)
Asian	28/158 (18%)
Black or African American	9/158 (6%)
White	102/158 (65%)
Mixed	7/158 (4%)
Other	11/158 (7%)
**Hispanic Origin**	15/155 (10%)
**U.S. Citizens**	116/157 (74%)
**Degree Being Pursued**	
High School	1/145 (1%)
Bachelor’s Degree	16/145 (11%)
Master’s Degree	19/145 (13%)
Doctorate	27/145 (19%)
Currently Practicing	72/145 (50%)
Other	10/145 (7%)
**Primary Field or Vocation**	
Medicine	73/158 (46%)
Public Health	23/158 (15%)
Nursing	18/158 (11%)
Basic Science	13/158 (8%)
International Development/Aid	5/158 (3%)
Health Policy	3/158 (2%)
Social Sciences	2/158 (1%)
Physicians’ Assistant	1/158 (1%)
Pharmacy	1/158 (1%)
Other	19/158 (12%)

Our survey asked users about past and future experiences in global health and global health ethics. See Table 2. Nearly two-thirds of open users have been abroad before for global health training or service, with a wide range of frequencies and durations. Among those who have been abroad before, only 52% reported having had any kind of global health ethics training; of these, 55% reported that this ethics training was directly relevant to short-term work abroad. Taken together 31% of users who have been abroad reported prior ethics training directly related to short-term work. When looking at prior global health ethics training and number of times abroad, individuals who had been abroad “more than 5 times” reported having had global health ethics nearly twice as often as individuals in the 1–2 and 3–5 groups and more than three times as often as individuals who had never been broad; this was statistically significant (Chi-square 13.8, p=0.003, for the four group comparison). Just over one-third of total users reported that this curriculum will be their only ethics training; nearly two thirds of users were planning an upcoming trip.

**Table 2:** **Prior global health and global health ethics experience** of users **(total N = 156, users do not have to answer all questions)**

**Have been abroad before**	**98/156 (63%)**
**Number of times abroad**	
1-2	31/97 (32%)
3-5	27/97 (28%)
More than 5	38/97 (39%)
**Average length**	
<4 weeks	36/95 (38%)
4-8 weeks	28/95 (29%)
8-12 weeks	9/95 (9%)
>12 weeks	22/95 (23%)
**Have had prior global health ethics training**	
*Overall*	61/151 (40%)
Abroad before	49/95 (52%)
Never abroad	12/56 (21%)
*By times abroad*	
1-2 times	13/31 (42%)
3-5 times	10/27 (37%)
More than 5 times	27/38 (71%)
**Prior ethics training was related to short-term work**	
*Overall*	34/62 (55%)
Abroad before	29/95 (31%)
Never abroad	12/53 (23%)
**Curriculum will be only ethics training**	50/145 (34%)
**Upcoming trip planned**	
Yes	99/153 (65%)
No	54/153 (35%)

To supplement and corroborate web statistics, we asked how users learned about the curriculum. Similar to web data, direct referrals from colleagues or via a user’s training program were most common (62%), followed by web search (28%) and web links (8%). Thirty-five users (24%) reported that their training program required them to take the curriculum.

Table 3 displays data from open user evaluation of individual cases in the curriculum. The most commonly evaluated case was “Developing Cultural Understanding” (N = 151). With the exception of the first three cases, approximately half of all users agreed or strongly agreed with the statement that the ethical issues presented were “new to me.” For all cases, more than 70% of users agreed or strongly agreed that the case gave them a strategy for dealing with the ethical issue in question. A large majority of users viewed the cases positively and would recommend them to a friend. Although negative feedback was infrequent in the open-ended response section, commonly expressed concerns included dissatisfaction with the frequency of answer choices involving consultation with a local supervisor or mentor; a need for greater depth in cases; and the focus of the cases on “medical” scenarios, as opposed to public health or engineering.

**Table 3:** Assessment of specific cases using a 5-point Likert scale (1 = Strongly Disagree, 5 = Strongly Agree) or yes/no as indicated

**Case (website sidebar order)**	**N**	**“Ethical issues were new to me”**	**“Gave me a strategy”**	**“Would recommend to a friend”**
		**Agree or strongly agree (Average score)**	**Agree or strongly agree (Average score)**	**Yes**
Developing Cultural Understanding	151	22%	81%	85%
		(2.51)	(3.87)	
Ensuring Personal Safety	110	35%	77%	85%
		(2.88)	(3.89)	
Exceeding Level of Training	96	32%	73%	80%
		(2.89)	(3.79)	
Ensuring Sustainable and Appropriate Benefits	88	51%	79%	91%
		(3.30)	(3.90)	
Addressing “Ancillary Benefits”	85	49%	85%	88%
		(3.24)	(4.00)	
Recognizing Burdens	77	43%	72%	88%
		(3.17)	(3.80)	
Shifting Resources	77	56%	79%	84%
		(3.36)	(3.82)	
Telling the “Truth”	86	52%	77%	88%
		(3.36)	(3.88)	
Selecting a Research Project	83	51%	78%	92%
		(3.26)	(3.86)	
Understanding Informed Consent	88	55%	79%	89%
		(3.27)	(3.90)	

## Discussion

The development and initial evaluation of http://ethicsandglobalhealth.org presented here is, to our knowledge, the first attempt to design and evaluate an online, widely accessible, introductory curriculum focused on ethical issues trainees might face in short-term training and service programs in global health. It was developed in direct response to increasing recognition of the ethical issues arising in such programs, emerging consensus around best practice guidelines, and a perceived need to translate these guidelines into an accessible format, especially for trainees or those with little prior global health experience. Our findings have important implications for ethics education related to short-term global health programs specifically and online ethics education more generally.

For instance, our data support the belief that more ethics training is needed for individuals traveling abroad for short-term global health programs. Less than one-third of users who have been abroad before report having had ethics training directly related to short-term work. Only after more than five trips abroad do a majority of individuals report having had ethics training. About one-quarter of users reported that this curriculum would be their only ethics training.

In addition, our data suggest that the curriculum is meeting its goal of wide accessibility and use. First, the curriculum is reaching a diverse range of fields, including medicine, public health, and nursing. Individuals of various nationalities are using the curriculum, and it is being disseminated via personal referral and through training programs, with a number of users locating the curriculum via web search. These observations encourage curriculum developers to consider ongoing direct dissemination of curricula to colleagues (perhaps including social media) and to use proven strategies to improve their curriculum’s ranking on Google and other search engines. Third, the curriculum content is generally well received based on responses to our Likert scale questions, with users generally perceiving that it offered both new content and new strategies for navigating ethical issues in this setting. Fourth, a number of programs appear to be requiring the curriculum before travel abroad, reinforcing the usefulness and perceived necessity of ethics education.

Despite this progress, work remains to meet our other curriculum goals. We were surprised, for example, at the average age of users (37 years) and that half were already practicing in their fields. While this suggests the curriculum may be effectively reaching those not in training programs, it might also suggest a need to better reach or target younger trainees. Similarly, although cases were well-received, a few cases (“Developing Cultural Understanding,” “Exceeding Level of Training,” and “Ensuring Personal Safety”) were perceived as less “new.” While these cases are arguably fundamental to global health training programs, future iterations of curriculum content might be able to cover these issues in more depth.

From a broader perspective, the curriculum at http://ethicsandglobalhealth.org is not the only online curriculum available. Other relevant online ethics resources that are freely available and directly related to short-term global health training (as opposed to global health ethics more generally) are shown in Table 4. Each has unique features. For example, the Global Health Education Consortium (GHEC) has teaching modules available in Spanish. Both GHEC and Unite for Sight integrate their ethics and professionalism teaching within broader global health topics. The University of British Columbia site has a quite comprehensive curriculum, including a detailed conceptual framework and pedagogical resources for instructors.

**Table 4:** Comparing freely available online curricula for short-term global health ethics

**Website**	**Broad conceptual framework**	**Primarily case-based**	**Includes specific cases or scenarios**	**Certificate available for completion**	**Part of global health curriculum**	**Some modules in Spanish**	**Notes**
**Global Health Education Consortium (GHEC) Teaching Modules**			**X**		**X**	**X**	· Self-navigation through PowerPoint
http://globalhealtheducation.org/modules/SitePages/Home.aspx							· Ethics integrated within "Working and Visiting in Low Resource Countries"
**Unite for Sight Volunteer Ethics and Online Professionalism Course**			**X**	**X (cost)**	**X**		· Self-navigation through text based modules
http://www.uniteforsight.org/international-volunteering/							· Focuses on professional behavior abroad
**Stanford Center for Innovation in Global Health and Johns Hopkins Berman Institute of Bioethics, “Ethical Challenges in Short-term Global Health Training”**							· Self-navigation through cases
http://ethicsandglobalhealth.org		**X**	**X**	**X (free)**			· Focuses on common scenarios
							· Employs multiple choice questions and real-time feedback
**University of British Columbia, “Ethics of International Engagement and Service-Learning Project”**							· Users navigate through various menus
http://ethicsofisl.ubc.ca/	**X**		**X**				· Includes cases, pedagogy, and philosophical analysis
							· Employs open-ended question and answers

Our curriculum, however, was designed to meet a particular niche in light of known advantages and disadvantages of online ethics education [41]. For example, some qualitative evidence suggests that teaching complex ethics concepts online is difficult [42]. We chose an online format for several reasons. First, our introductory curriculum does not teach complex concepts but instead introduces individuals with little or no prior training to a broad range of issues. Second, we wanted the introductory curriculum to be free and widely available, without requiring a login and with accessibility at all times. Third, an online site allows training programs and educators to tailor the curriculum for their particular needs, including within more comprehensive ethics curricula [43]. Our use of real life cases, for example, fits well within current models of medical ethics education [44] and the high prevalence case- or problem-based methods for teaching ethics (e.g., at U.S. medical schools [45]). Fourth, because some trainees organize and participate in short-term programs outside their training institution, we wanted the curriculum to be available independent of specific institutions or programs.

Our findings must be interpreted in light of several limitations. First, web statistics can be difficult to interpret, likely overestimate the number of unique visitors, and cannot track whether or to what extent visitors complete site content. The open user group may be subject to ascertainment or sample selection bias. For example, open users searching for a curriculum online may be less likely to have had ethics training in the past and hence desire to search for it. This prevents us from making broad claims about the general population of individuals who go abroad for short-term global health training. The use of open user data – unlike standard pre- and post-test methods [46,47] – might nevertheless better represent the real-world and intended use of online curricula, which is particularly important for the heterogeneous group of individuals who travel abroad for short-term global health training and service. Second, because our curriculum is introductory, it cannot address ethical issues specific to every situation, such as unique issues that might arise within specific medical specialties (e.g., pediatrics or obstetrics) or specific locations (e.g., global health programs which include underserved areas in one’s own country). Finally, although WEIGHT and CUGH include individuals and institutions in LMICs, thereby informing the process, content was determined and evaluated predominantly by individuals from high income countries. This suggests a need to develop and implement future curricula with greater input from those abroad, especially those in LMICs or the “global South.”

## Conclusions

In summary, we developed a widely accessible, online introductory ethics curriculum for short-term training and service programs in global health. Our data suggest that a number of individuals go abroad without first receiving ethics training specifically related to short-term work. This could be related in part to the relative lack of available ethics education curricula until recently. Open user data suggest that our curriculum is reaching a diverse segment of its target audience. Future evaluations will focus on how well the curriculum increases knowledge of specific ethical issues arising in short-term global health training programs. In addition, a need exists to further develop and integrate this introductory curriculum into more comprehensive curricula; to demonstrate real behavioral changes among those going abroad; and to evaluate the effect such curricula have on the conduct of training programs on-the-ground at host sites. Our introductory curriculum is meant to introduce some of the ethical issues in short-term global health training, not replace more comprehensive courses or in-depth discussion of ethical concepts. This curriculum can therefore serve as a resource for global health training programs to prepare those involved for the ethical issues inherent in such work.

## Competing interests

The authors declare that they have no competing interests.

## Authors’ contributions

MD made substantial contributions to the conception and design of the study; the acquisition of data; the drafting of the manuscript; and critical revisions of the final version. JR made substantial contributions to the conception and design of the study; the acquisition of data; and the drafting of the manuscript. SH made substantial contributions to the acquisition of data and the drafting of the manuscript. MB made substantial contributions to the conception and design of the study and critical revisions of the final version. JS made substantial contributions to the conception and design of the study; the drafting of the manuscript; and critical revisions of the final version. All authors approved the final version of the manuscript.

## Supplementary Material

Additional file 1: Appendix 1 Open user anonymous survey. Click here for file

Additional file 2: Appendix 2 Survey following each case. Click here for file
