# Supplementary material for: An ethics curriculum for short-term global health trainees
Source: Global Health. 2013 Feb 14;9:5. doi: 10.1186/1744-8603-9-5 (PMC3598721; doi:10.1186/1744-8603-9-5)
Supplement: Additional file 1: Appendix 1 — Open user anonymous survey. [file 1744-8603-9-5-S1.doc]

**Appendix 1. Open user anonymous survey.**

|  | [**About You**](http://www.surveymonkey.com/MySurvey_EditPage.aspx?sm=qsMURyOmhU15ZI%2FeLC3gDTsb3qHdxzMXYhRtK91jOMCk6qDXfJZ1XFaekWQ1Ur9U&TB_iframe=true&height=450&width=650)  1. Are you male or female?   | Male  Female | | --- |   2. How old are you?    3. What is your race/ethnicity?   | American Indian or Alaska Native  Asian  Black or African American  Native Hawaiian or Other Pacific Islander  White  Mixed | | --- | | Other (please specify) |   4. Are you of Hispanic, Latino, or Spanish Origin?   | No  Yes – Mexican or Mexican-American, Chicano  Yes – Puerto Rican  Yes – Cuban | | --- | | Yes - Another (please specify) |   5. What is your nation of citizenship?   | United States | | --- | | Other (please specify) |   6. What level of education have you achieved up till now? (Select the highest achieved)   | High School  Vocational School  Bachelor’s Degree/University  Master’s Degree  Doctorate  None of the above | | --- |   7. If you are currently in a degree granting program, what degree are you pursuing?   | High School  Bachelor’s Degree/University  Master’s Degree  Doctorate  Not applicable (currently practicing in my field) | | --- | | Other (please specify) |   8. What specialty or field do you consider your main vocation?   | Basic Science  Engineering  Health Policy  International Development/Aid  Medicine  Nursing  Pharmacy  Physical Therapy  Physician’s Assistant  Public Health  Social Sciences | | --- | | Other (please specify) | | |  |
| --- | --- | --- | --- | --- | --- | --- | --- | --- | --- | --- | --- | --- | --- | --- | --- |
| [**About Your Prior Experience in Global Health**](http://www.surveymonkey.com/MySurvey_EditPage.aspx?sm=qsMURyOmhU15ZI%2FeLC3gDan1DxIsPq405XvMm%2FFjPm7KsuWP3Q4FCV18Jbzk548i&TB_iframe=true&height=450&width=650)  9. Have you been abroad previously for global health training or service?   | Yes  No | | --- | | |  | |
| 10. How many times have you been abroad previously for global health training or service purposes? (If 0, skip to question 13.)   | 0  1-2  3-5  More than 5 | | --- |   11. On average, how long were these prior trips abroad for global health training or service purposes?   | Less than 4 weeks  4-8 weeks  8-12 weeks  Greater than 12 weeks | | --- |   12. In what regions have you previously traveled for global health training or service purposes? Choose all that apply.   | Africa  Americas  Eastern Mediterranean  Europe  Southeast Asia  Western Pacific | | --- | | Other (please specify) | | | | |

| [**About Your Next Global Health Training or Service Program**](http://www.surveymonkey.com/MySurvey_EditPage.aspx?sm=qsMURyOmhU15ZI%2FeLC3gDan1DxIsPq405XvMm%2FFjPm4H14vG0SNwXDJZJqn8fYMl&TB_iframe=true&height=450&width=650)  13. Do you have a future short-term global health training or service program planned? (If “No,” skip to question 16.)   | Yes  No | | --- | |
| --- | --- |

| [**About Your Next Global Health Training or Service Program**](http://www.surveymonkey.com/MySurvey_EditPage.aspx?sm=qsMURyOmhU15ZI%2FeLC3gDan1DxIsPq405XvMm%2FFjPm5lfFv4oYjs9Ab4ytwVRtp1&TB_iframe=true&height=450&width=650)  14. Where will this next training or service program be? Choose all that apply.   | Africa  Americas  Eastern Mediterranean  Europe  Southeast Asia  Western Pacific | | --- | | Other (please specify) |     15. How long will this future training or service program be?   | Less than 4 weeks  4-8 weeks  8-12 weeks  Greater than 12 weeks | | --- | | | | | |
| --- | --- | --- | --- | --- | --- | --- | --- |
|  | [**About Your Prior Experience in Global Health Ethics**](http://www.surveymonkey.com/MySurvey_EditPage.aspx?sm=qsMURyOmhU15ZI%2FeLC3gDan1DxIsPq405XvMm%2FFjPm4jdiVTPWBD4YMVmu%2F3AK6g&TB_iframe=true&height=450&width=650)  16. Have you had prior global health ethics training?   | Yes  No | | --- | | | |  |
|  |  |  |  | |

| 17. Was this prior ethics training directly relevant to short-term training or service abroad?   | Yes  No | | --- | |  |
| --- | --- | --- |
| [**About this Introductory Curriculum**](http://www.surveymonkey.com/MySurvey_EditPage.aspx?sm=qsMURyOmhU15ZI%2FeLC3gDan1DxIsPq405XvMm%2FFjPm7xOpEjl2rBDqE6b3rCCU4J&TB_iframe=true&height=450&width=650)  18. Will completion of this curriculum be your only ethics training prior to your next short-term training or service program abroad?   | Yes  No  Don't know - unsure what program is planning  Don't know - no program planned | | --- |   19. Is completion of this curriculum required by your training or service program?   | Yes  No  Not applicable - no program planned | | --- |   20. How did you learn about this curriculum? Choose all that apply.   | Referral from a friend or colleague  Through my training program  Web search | | --- | | Other (please specify) | | |

|  |  |
| --- | --- |

|  |
| --- |
