# Supplementary material for: An ethics curriculum for short-term global health trainees
Source: Global Health. 2013 Feb 14;9:5. doi: 10.1186/1744-8603-9-5 (PMC3598721; doi:10.1186/1744-8603-9-5)
Supplement: Additional file 2: Appendix 2 — Survey following each case. [file 1744-8603-9-5-S2.doc]

**Appendix 2. Survey following each case.**

1. Overall this case was useful preparation for my training abroad.

| Strongly Disagree | Disagree | Neither agree/disagree | Agree | Strongly Agree |
| --- | --- | --- | --- | --- |

2. The ethical issues presented in this case...

|  | **Strongly Disagree** | **Disagree** | **Neither agree/disagree** | **Agree** | **Strongly Agree** |
| --- | --- | --- | --- | --- | --- |
| ...were new to me. |  |  |  |  |  |
| ...gave me a strategy for dealing with these ethical issues. |  |  |  |  |  |
| ...increased my confidence in dealing with these ethical issues. |  |  |  |  |  |
| ...will improve how I behave during my next training program abroad. |  |  |  |  |  |

3. Why did you do this case?

| Requirement of my program  Thought it would be of personal benefit  Both |
| --- |

**4. Do you have any recommendations for changing this case?**
Comment on structural (e.g., web design, font, video performance, etc.) and/or substantive (e.g., level of detail, concreteness of strategies, representation of issue) concerns.

5. Would you recommend this case to a friend?

| YesNo |
| --- |
